# Dynamic analysis of critical maternal complications in tertiary hospitals in Wuxi: A study based on four years of monitoring data

**DOI:** 10.1371/journal.pone.0351670

**Published:** 2026-06-12

**Authors:** Ye Xu, Peimin Hua, Ye Shen

**Affiliations:** 1 Department of Community Health, Wuxi Maternity and Child Health Care Hospital, Wuxi, Jiangsu Province, China; 2 Department of Obstetrics, Wuxi Maternity and Child Health Care Hospital, Wuxi, Jiangsu Province, China; 3 Reproductive Health and Family Planning Department Wuxi Maternity and Child Health Care Hospital, Wuxi, Jiangsu Province, China; Xiangya Hospital Central South University, CHINA

## Abstract

**Background:**

Severe maternal morbidity (SMM) is a significant public health concern. This study analyzed the incidence, trends, causes, and pregnancy outcomes of SMM in Wuxi to inform future clinical and public health strategies.

**Methods:**

A retrospective analysis was conducted on 315 critical maternal cases identified from 156,435 deliveries in Wuxi between October 1, 2020, and September 30, 2024. Data were extracted from a citywide near-miss maternal surveillance system. Statistical analyses were performed using SPSS 25.0, employing chi-square tests and Cochran-Armitage trend tests to evaluate trends, and chi-square tests for comparisons between groups.

**Results:**

The overall incidence of SMM was 0.20%. Initially, this rate remained stable at 0.19% across the first three cycles (P > 0.05); however, it significantly increased to 0.24% during the cycle from October 2023 to September 2024 (χ² = 5.24, P = 0.02). This increase was closely associated with a rise in the proportion of women of advanced maternal age (≥35 years), which reached 26.03% (χ² = 11.76, P = 0.001). Over time, the distribution of risk levels shifted. Initially, the high-risk group was dominant (63.29%), but in recent cycles, the moderate-risk group became more prominent (64.44%). The moderate-risk group was associated with a higher rate of adverse outcomes (25.00–25.71%) compared to the high-risk group (17.11–20.69%; χ² = 10.83, P = 0.01). Direct obstetric factors were the primary causes, accounting for 79.05% of cases, with obstetric hemorrhage being the most prevalent (53.97%). In contrast, the proportion of cases attributable to indirect obstetric factors increased from 17.81% to 26.67%, primarily due to heart disease and infectious diseases.

**Conclusion:**

Improving maternal safety involves dynamic risk assessments, tiered referrals for moderate-risk pregnancies, better multidisciplinary management of complications, optimized emergency responses in primary care, and refined regional referral systems to reduce preventable SMM and mortality.

## Introduction

Improving maternal health remains a global priority, with Sustainable Development Goal 3.1 (SDG 3.1) explicitly aiming to reduce the global maternal mortality ratio (MMR) [1]. Although the WHO estimates a 34% decline in the global MMR between 2000 and 2020, approximately 260,000 maternal deaths still occurred annually as of 2020, with 70% concentrated in sub-Saharan Africa and South Asia [2]. Nevertheless, even high-income countries face persistent challenges; for example, the United States has the highest MMR among developed nations, a disparity largely driven by racial inequities and inadequate access to timely care [3,4]. Beyond mortality, Severe maternal morbidity (SMM) presents a significant burden. Beyond mortality, severe maternal morbidity (SMM) represents a profound threat to women’s health and well-being. Women who experience SMM face not only an immediate risk of death but also long-term physical and psychological sequelae, including chronic organ dysfunction, post-traumatic stress, and reduced quality of life. A Swedish national cohort study found that SMM was associated with a 17% lower likelihood of subsequent childbirth, underscoring the enduring impact of these events on women’s lives. Globally, SMM occurs 10–20 times more frequently than maternal death, making it a more sensitive indicator of obstetric care quality [5]. Globally, SMM occurs 10–20 times more frequently than maternal death, making it a more sensitive indicator of obstetric care quality [6,7]. These findings underscore the urgent need for more refined and proactive strategies that move beyond conventional mortality-focused interventions to comprehensively safeguard maternal health.

In response to these limitations, the concept of Maternal Near Miss (MNM) has emerged as a key complementary indicator. The World Health Organization (WHO) defines an MNM as a woman who nearly died but survived a complication that occurred during pregnancy, childbirth, or within 42 days of the termination of pregnancy [8]. These survivors offer a critical clinical window, serving as sentinel events that reveal systemic weaknesses in obstetric care that are often invisible in mortality data alone [8,9]. For instance, a systematic review of 30 studies in low- and middle-income countries identified MNM as a stronger predictor of healthcare system weaknesses than MMR, particularly in resource-limited settings [10,11]. To standardize global case identification and analysis, the WHO developed a set of unified, practical diagnostic criteria and a comprehensive indicator system for obstetric quality assessment in 2009 [8]. This framework, based on organ dysfunction, enables robust cross-institutional and international comparisons, transforming MNM audits into a powerful quality improvement tool [8]. Studies implementing the WHO MNM standards have demonstrated tangible impacts: a multicenter study in Brazil found that systematic MNM audits reduced severe postpartum hemorrhage rates by 28% within two years [12,13], while a similar initiative in India improved timely access to emergency cesarean sections by 35% [14,15].

In China, coordinated national efforts have significantly reduced the MMR from 88.8 to 14.3 per 100,000 live births between 1990 and 2024, positioning the country among the top performers in terms of maternal health [16]. Key strategies include the establishment of 3,491 critical maternal care centers, universal prenatal care coverage exceeding 90%, and the integration of maternal health into the “Healthy China 2030” agenda [16]. However, significant regional disparities persist: the MMR in western China remains 2.3 times higher than in the eastern provinces, and rural areas face ongoing challenges with healthcare access and specialist availability [17]. Consequently, the implementation of WHO’s MNM methodology at the sub-national level has become a focus of health services research in China [18,19]. Several provinces, including Guangdong and Zhejiang, have piloted MNM surveillance systems, revealing that hypertensive disorders of pregnancy and postpartum hemorrhage account for 60–70% of local MNM cases [20]. To address this need, the Jiangsu Provincial Health Commission issued the “Implementation Plan for the Analysis of Maternal Near Miss Cases in Jiangsu Province” in 2020, establishing standardized provincial screening criteria and a comprehensive reporting and review system [20]. This system mandates multicenter data collection and root-cause analysis of MNM events, aligning with national guidelines for maternal risk stratification using a five-color classification system (green, yellow, orange, red, and purple) [19,21]. Despite this provincial framework, critical knowledge gaps remain at the municipal level in Jiangsu. While existing studies have focused on provincial-level epidemiology or single-center experiences, no systematic investigation has analyzed the specific risk profiles, etiological patterns, or outcomes from multidisciplinary case reviews of SMM in Wuxi to date. Similar municipal studies in China have demonstrated the value of localized data. An analysis in Shanghai identified gestational diabetes and advanced maternal age as key predictors of MNM, leading to targeted prenatal intervention programs [22].

To address this gap, the present study aimed to leverage existing municipal surveillance systems to conduct a retrospective analysis of all reported SMM cases in Wuxi from October 1, 2020, to September 30, 2024. The specific objectives of this study are to describe the incidence and epidemiological characteristics of SMM cases in the region, including age distribution and risk factor profiles. The main causes and associated factors of SMM were identified, with a focus on modifiable clinical and system-level determinants. By achieving these objectives, this study will provide evidence-based insights to directly inform the development of clinical guidelines and public health strategies in Wuxi and similar urban settings in China. The findings will contribute to ongoing efforts to reduce preventable maternal deaths and severe morbidity, aligning with both SDG 3.1 and China’s “Healthy China 2030” goal.

Wuxi is a major city in Jiangsu Province, eastern China, with a population of approximately 7.5 million. It serves as a regional referral center for high-risk pregnancies in the Yangtze River Delta, supported by a network of tertiary hospitals and a well-established maternal surveillance system. In this study, we adopt the term “severe maternal morbidity (SMM)” to refer to critical maternal cases meeting the WHO-recommended organ dysfunction-based criteria and Jiangsu provincial screening standards. The term “maternal near miss (MNM)” is used only when referring to the WHO conceptual framework in the literature.

## Methods

### Study design and setting

This retrospective study analyzed critical maternal cases identified from all deliveries in Wuxi City between October 1, 2020, and September 30, 2024. The cycle boundaries (October to September) were chosen to align with the fiscal year of the Wuxi municipal health reporting system, facilitating administrative consistency and data aggregation across reporting institutions. The observation period spanned from pregnancy to 42 days postpartum.

### Data source and participants

All critical maternal cases and related data used in this study were sourced from pregnant women who underwent prenatal checkups and delivered in Wuxi City. A total of 23 obstetric institutions participate in the municipal surveillance system, including 8 tertiary hospitals and 15 secondary hospitals. In accordance with the Wuxi maternal referral guidelines, all suspected or confirmed severe maternal cases at secondary hospitals must be transferred to tertiary hospitals for definitive management. Therefore, the 315 cases identified in this study, all from tertiary hospitals, are considered to represent the complete spectrum of SMM in the region. The observation period spanned from pregnancy to 42 days postpartum. Since 2020, the Jiangsu Provincial Health Commission has implemented the Implementation Plan for Critical Maternal Cases Analysis in Jiangsu Province and established screening criteria for critical maternal cases. All cases that met these criteria were reported. Quarterly, all obstetric institutions across the city report critical maternal cases to the municipal perinatal care collaboration group in accordance with established standards. The statistical period for critical maternal cases spans October 1, 2020, to September 30, 2024. The cycle boundaries (October to September) were chosen to align with the fiscal year of the Wuxi municipal health reporting system, facilitating administrative consistency and data aggregation across reporting institutions. All reported critical maternal cases underwent analyses and reviews at the hospital and county levels. The municipal Perinatal Health Care Collaborative Group invited multidisciplinary experts from the provincial and municipal levels to analyze and review the reported cases.

Research data were obtained from the dedicated database established by the Jiangsu Provincial Health Commission under the Critical Maternal Case Analysis Implementation Plan. The data were accessed on 17/10/2024 for research purposes.

### Definitions and criteria

Based on the WHO-recommended diagnostic criteria for critical maternal cases and Jiangsu provincial standards [23], pregnant women meeting any of the following conditions were included in the monitoring scope: (1) severe obstetric complications, including severe preeclampsia/eclampsia, severe postpartum hemorrhage (blood loss ≥ 1000mL or requiring blood transfusion), amniotic fluid embolism, uterine rupture, and acute fatty liver of pregnancy. (2) Severe internal/surgical comorbidities, including cardiac disease during pregnancy, sepsis, severe pneumonia, acute pancreatitis, and autoimmune diseases. (3) Organ function support therapy, including mechanical ventilation for ≥ 60 min, cardiopulmonary resuscitation, renal dialysis, and hysterectomy.

All reported cases underwent a three-tiered review by hospital, county, and city perinatal care collaboration groups. Final confirmation is performed by provincial/municipal multidisciplinary expert teams (obstetrics, critical care medicine, anesthesiology, cardiology, etc.) to ensure the accuracy and completeness of case identification.

**Fig 1:**
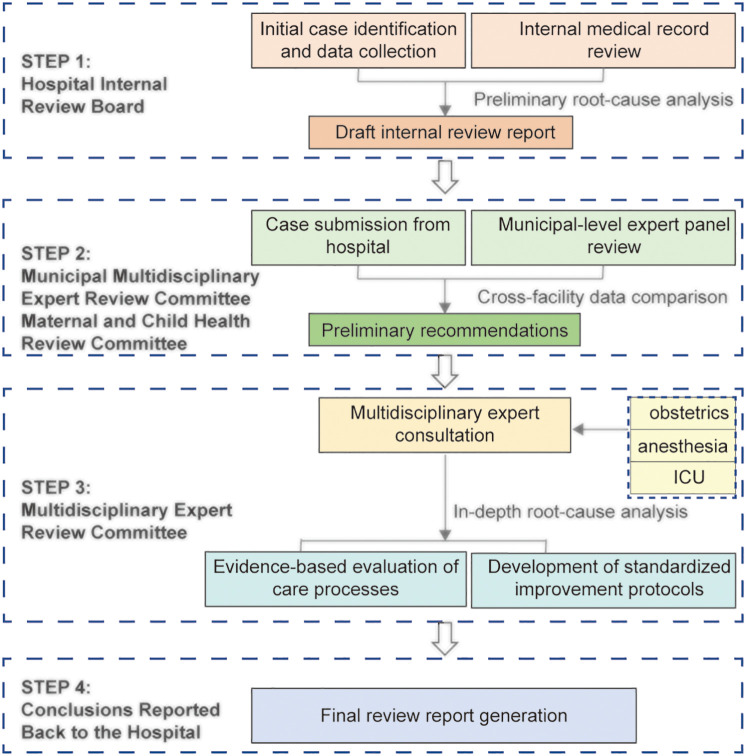
Critical Pregnant Women Review Flowchart.

The review of critical maternal cases and associated risk assessments is guided by rigorous scientific principles. The primary objective of the program is to enhance maternal safety by systematically analyzing cases of severe obstetric complications to identify weaknesses in the delivery of medical services. These reviews typically adopt a framework similar to maternal death surveillance, involving multi-tiered, multidisciplinary expert committees that conduct in-depth assessments of each case (Fig 1). Before conducting these reviews, the data were accessed on 17/10/2024 for research purposes to enable systematic analysis of weaknesses in care delivery. Risk assessment was the core of this review. It requires not only a precise clinical diagnosis but also the identification of weaknesses throughout the entire chain of care, from prevention and early detection to intervention and effective referrals. This structured process translates lessons from individual cases into strategies for systemic improvements. This provides an evidence-based foundation for developing targeted interventions, optimizing resource allocation, and refining the maternal critical care network, ultimately reducing preventable SMM and mortality.

Maternal risk was graded using Jiangsu Province’s standardized five-color system, which aligns with national guidelines: green (low risk, normal pregnancy), yellow (moderate risk, e.g., gestational diabetes without complications), orange (higher risk, e.g., preeclampsia or cardiac disease with mild symptoms), red (high risk, e.g., severe organ dysfunction or life-threatening conditions), and purple (infectious diseases, e.g., active tuberculosis or viral hepatitis). In this study, purple-coded cases were classified under “indirect obstetric factors” (specifically infectious diseases) and are not presented as a separate category in the risk distribution tables due to their small number (n = 8) and to maintain consistency with the direct/indirect etiology framework.

The following data quality control measures were implemented: (1) quarterly citywide audits of reports from all obstetric institutions, ensuring an underreporting rate below 5%; (2) logical checks for data anomalies; cases with mismatched diagnoses and interventions were returned to the reporting institutions for verification and correction; and (3) random re-evaluation of 20% of all cases by the municipal expert panel, achieving a diagnostic consistency rate exceeding 95%. All quality control procedures were performed on the dataset accessed on 17/10/2024 for research purposes.

### Data collection

We used a standardized form to extract data from the electronic medical records. The following variables were included: (1) maternal demographics: age, place of residence (urban/rural), and adequacy of prenatal care; (2) obstetric characteristics: gravidity (total number of pregnancies), parity (number of deliveries), gestational age at delivery, and mode of delivery; (3) clinical diagnosis: primary obstetric cause, categorized as direct or indirect, and coded according to the ICD-10 system; (4) clinical interventions: respiratory support (defined as mechanical ventilation ≥60 min), blood transfusion, cardiopulmonary resuscitation, renal dialysis, and peripartum hysterectomy; (5) pregnancy outcomes: live birth versus stillbirth, and gestational age at termination; and (6) adverse maternal outcomes: a composite endpoint including maternal death, intensive care unit (ICU) admission or multiple organ dysfunction syndrome (MODS).

Classification of pregnancy-related complications: Specific conditions mentioned in the literature, such as preeclampsia and eclampsia, were captured under the broader category of “gestational hypertensive disorders” within direct obstetric factors. This category includes all cases of severe preeclampsia, eclampsia, and chronic hypertension with superimposed preeclampsia that met the provincial criteria for critical maternal case reporting. Iron deficiency anemia was not included as a separate variable because it is not part of the provincial screening criteria unless it reaches severity requiring blood transfusion (in which case it would be captured under “obstetric hemorrhage” or “blood transfusion” as an intervention). High-altitude pregnancy did not apply to this study population, as Wuxi is located in the Yangtze River Delta at an elevation of approximately 10 meters above sea level, and no cases of recent migration from high-altitude regions were documented in the surveillance database. Data extraction commenced immediately after the dataset was accessed on 17/10/2024 for research purposes.

Data extraction commenced immediately after the dataset was accessed on 17/10/2024 for research purposes.

Potential influencing factors: The surveillance database routinely recorded obstetric history variables, including gravidity and parity. These variables were extracted and considered in the descriptive analysis of maternal characteristics. However, maternal marital status at the time of pregnancy was not captured by the surveillance system, as this information is not routinely collected in the clinical records of the participating hospitals under the provincial reporting protocol. Therefore, marital status could not be analyzed as a potential factor impacting pregnancy outcomes or postpartum health in this study.

### Statistical analysis

Data analysis was conducted using SPSS 25.0 software, employing descriptive statistics. Continuous variables are presented as mean ± standard deviation or median (interquartile range), based on their normal or non-normal distribution. Categorical variables are expressed as numbers (percentages). Temporal trends in incidence rates across the four cycles were assessed using the chi-square test for trend (Cochran-Armitage test for binary outcomes), which is appropriate for detecting monotonic trends across ordered groups. Given the small number of time points (n = 4) and the primary interest in identifying whether the final cycle differed significantly from the preceding stable period, we supplemented the trend test with pairwise comparisons between each cycle and the previous one using the chi-square test, with Bonferroni correction for multiple comparisons. This approach balances simplicity with the ability to detect the specific point of change observed in the data. Intergroup comparisons were performed using the chi-square test or Fisher’s exact test, as appropriate. Univariate analyses were performed using chi-square tests; because the study was descriptive, multivariate regression was not applied. A two-sided P-value < 0.05 was considered statistically significant. For multiple comparisons, the significance level was adjusted post hoc using the Bonferroni method to control the family-wise error rate.

### Ethics approval

Ethical approval for the Wuxi Maternal Near-Miss Surveillance System was granted by the Ethics Committee of Wuxi Maternity and Child Health Care Hospital (No: YLSL2025-0612-057). As this study involved a retrospective analysis of existing surveillance data without the direct involvement of human subjects, collection of personal identifiers, or risk to participants, the requirement for informed consent was waived by the ethics committee. All data were anonymized before analysis to ensure patient confidentiality.

## Results

### Incidence and temporal trends of severe maternal morbidity

**Fig 2:**
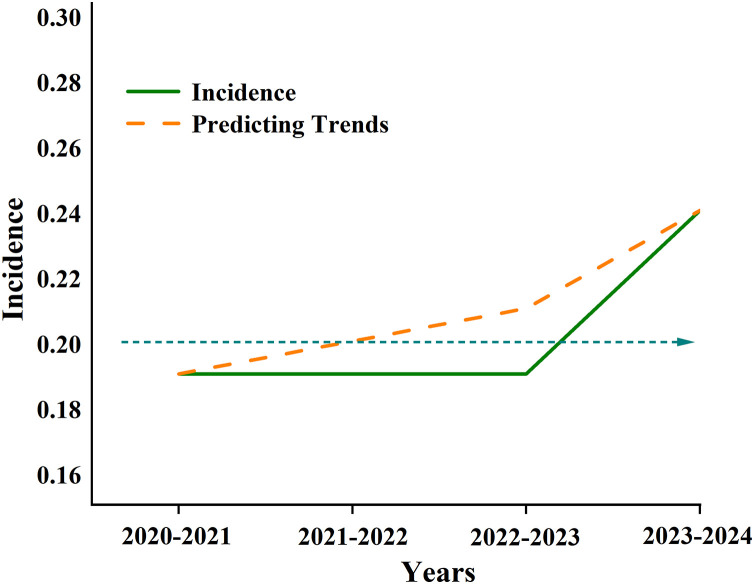
Trend in the Incidence of Critical Maternal Complications.

During the 4-year surveillance period (October 1, 2020, to September 30, 2024), 315 cases of SMM were identified among 156,435 deliveries in Wuxi tertiary hospitals, yielding an overall incidence of 0.20%. The temporal pattern exhibited three stable phases, followed by a significant surge in the final phase (Table 1; Fig 2). The incidence remained consistently at 0.19% across the first three surveillance cycles (October 2020–September 2023), with no statistically significant differences between the cycles (χ² = 0.05–0.18, all P > 0.05). In the final cycle (October 2023–September 2024), the incidence increased significantly to 0.24% (χ² = 5.24, P = 0.02). This upward trend was significantly associated with the rising proportion of women of advanced maternal age (≥35 years), which progressively increased from 21.52% in the first cycle to 32.22% in the final cycle (χ² = 11.76, P = 0.001) (Table 2).

**Table 1:** Combined Table of Incidence and Age Distribution of Critical Maternal Cases.

Monitoring Period	Total Deliveries (cases)	Number of Critically Ill Pregnant Women (cases)	Critically Ill Maternal Cases Incidence Rate (%)	Comparison with Previous Period Incidence Rate (χ² Value/P Value)
01/10/2020 − 30/09/2021	41,321	79	0.19	
01/10/2021 − 30/09/2022	40,243	76	0.19	χ² = 0.05/ P = 0.82
01/10/2022 − 30/09/2023	36,842	70	0.19	χ² = 0.18/ P = 0.67
01/10/2023 − 30/09/2024	38,029	90	0.24	χ² = 5.24/ P = 0.02
Total	156435	315	0.20	

**Table 2:** Incidence Rates of Critical Pregnancy Complications by Age Group in Wuxi City.

Monitoring Period	Age Distribution (n, %)	Comparison with previous cycle (χ²/P value)
01/10/2020 − 30/09/2021	<35: 62 (78.48); ≥ 35: 17 (21.52)	χ² = 3.52/P = 0.06
01/10/2021 − 30/09/2022	<35: 58 (76.32); ≥ 35: 18 (23.68)	χ² = 3.91/P = 0.04
01/10/2022 − 30/09/2023	<35: 52 (74.29); ≥ 35: 18 (25.71)	χ² = 4.37/P = 0.03
01/10/2023 − 30/09/2024	<35: 61 (67.78); ≥ 35: 29 (32.22)	χ² = 5.89/P = 0.01
Total	<35: 233 (73.97); ≥ 35: 82 (26.03)	χ² = 11.76/P = 0.001

The χ² value in each row compares the age distribution of the current monitoring cycle with that of the immediately preceding cycle. The first cycle has no preceding cycle, so the cell is marked as " –.” The χ² value in the " Total " row compares the first cycle (2020–2021) with the last cycle (2023–2024).

### Dynamic evolution of pregnancy risk stratification

**Fig 3:**
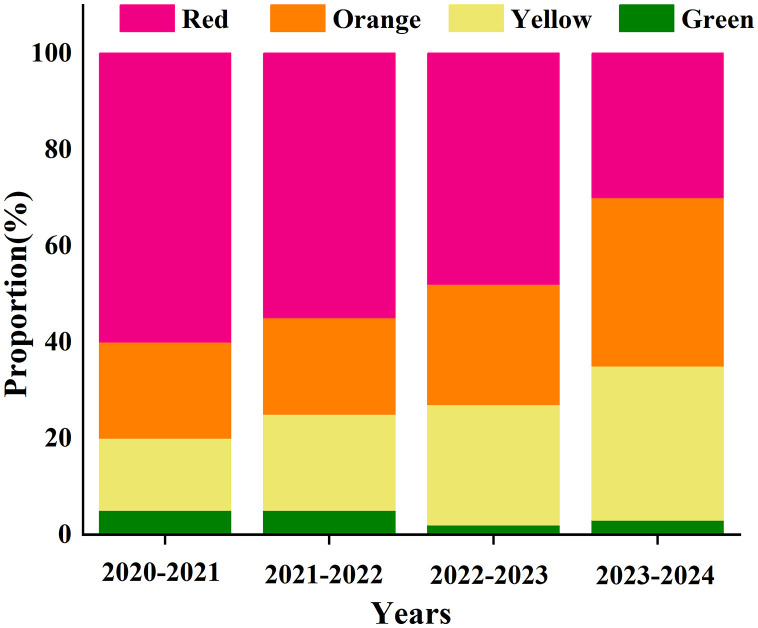
Evolution of Risk-Level Distribution. Risk levels are ordered from low to high: green, yellow, orange, red. Green denotes low-risk zones; yellow and orange indicate moderate-risk zones; red signifies high-risk zones.

The distribution of risk levels exhibited significant temporal heterogeneity across the surveillance cycles (χ² = 22.48–42.37, P < 0.001) (Table 3, Fig 3). In the initial monitoring period (October 2020–September 2021), high-risk (red-level) admissions predominated at 63.29% (50/79 cases), whereas low-risk (green) cases accounted for only 3.80% (3/79 cases). During the middle phase (October 2021–September 2023), a progressive risk de-escalation emerged: red-level proportions declined annually from 57.89% (44/76) to 50.00% (35/70), whereas moderate-risk categories demonstrated consistent expansion—yellow-level cases increased from 19.74% (15/76) to 27.14% (19/70), and orange-level cases rose from 17.11% (13/76) to 21.43% (15/70). This transition culminated in the final cycle (October 2023–September 2024), when combined moderate-risk cases (yellow + orange) became the dominant cohort for the first time, comprising 64.44% (58/90) of admissions and surpassing red-level cases (32.22%, 29/90). Notably, low-risk (green) cases remained marginal throughout, never exceeding 5.26% of the cohort in any period.

**Table 3:** Distribution and Dynamic Changes in Risk Levels for Critical Maternal Admissions.

Monitoring Period	Risk Level	Number of Critical Cases (n)	Proportion of Critical Cases in Same Period (%)	Year-on-Year Change Rate (%)	Adverse Outcomes by Risk Level (Number of Cases/Incidence Rate)
01/10/2020 − 30/09/2021	Green	3	3.80	–	0/0.00%
	Yellow	14	17.72	–	4/28.57%
	Orange	12	15.19	–	3/25.00%
	Red	50	63.29	–	8/16.00%
01/10/2021 − 30/09/2022	Green	4	5.26	+38.42	0/0.00%
	Yellow	15	19.74	+11.40	4/26.67%
	Orange	13	17.11	+12.64	4/30.77%
	Red	44	57.89	−8.55	7/15.91%
01/10/2022 − 30/09/2023	Green	1	1.43	−71.29	0/0.00%
	Yellow	19	27.14	+37.48	5/26.32%
	Orange	15	21.43	+25.25	5/33.33%
	Red	35	50.00	−13.63	6/17.14%
01/10/2023 − 30/09/2024	Green	3	0.24	+131.47	1/33.33%
	Yellow	28	31.11	+14.63	6/21.43%
	Orange	30	33.33	+55.53	7/23.33%
	Red	29	32.22	−35.56	6/20.69%
Total	Green	11	3.28	–	1/9.09%
	Yellow	76	22.69	–	19/25.00%
	Orange	70	20.90	–	18/25.71%
	Red	158	47.16	–	28/17.72%
Moderate-risk (Yellow+Orange) vs. High-risk (Red)		146 vs 158	43.59% vs 47.16%		37/25.34% vs 28/17.72% (χ² = 10.83, P = 0.01)

Critically, this risk redistribution was accompanied by a persistent paradox in maternal outcome. Despite risk stratification logic, moderate-risk groups (yellow and orange) exhibited consistently higher adverse outcome rates (25.00%–25.71%) than the high-risk (red) group (15.91% − 20.69%) across all surveillance cycles (χ² = 10.83, P = 0.01) (Table 3). This counterintuitive gradient was most pronounced in 2022–2023, when orange-level cases reached their peak adverse outcome rate of 33.33% (5/15), nearly double that of red-level cases (17.14%, 6/35). These findings suggest that conventional risk classification may inadequately capture the dynamic complexity of critical maternal morbidity, potentially reflecting either the preventive impact of intensified surveillance for red-tagged patients or under-recognition of deterioration mechanisms in moderate-risk cohorts.

### Etiological distribution and clinical outcomes

Direct obstetric factors accounted for 79.05% (249/315) of critical maternal cases, significantly exceeding indirect obstetric factors (20.95%, 66/315) in the overall distribution (χ² = 89.62, P < 0.001). Within the direct obstetric factors category, significant heterogeneity existed across the three subcategories (obstetric hemorrhage, hypertensive disorders, and amniotic fluid embolism) in both their proportional distribution (χ² = 82.45, P = 0.001, comparing the number of cases across subcategories) and their adverse outcome rates) (χ² = 14.79, P = 0.001, comparing the proportion of adverse outcomes across subcategories). Obstetric hemorrhage constituted the majority (53.97%, 170/315) with an adverse outcome rate of 21.08% (35/166), while hypertensive disorders of pregnancy (including severe preeclampsia, eclampsia, and chronic hypertension with superimposed preeclampsia) comprised 23.81% (75/315) with an adverse outcome rate of 18.67%. Most strikingly, amniotic fluid embolism, despite its rarity (1.27%, 4/315), exhibited a catastrophic adverse outcome rate of 75.00% (3/4). Among indirect factors, cardiovascular disease was the leading cause (6.03%, 19/315), with a 26.32% adverse outcome rate (5/19). Infectious diseases (4.13%, 13/315) and pneumonia (0.95%, 3/315) demonstrated substantial morbidity, with adverse outcome rates of 25.00% and 33.33%, respectively. Acute pancreatitis (1.90%, 6/315) contributed to a 16.67% adverse outcome rate (1/6). The residual “Other” category, encompassing autoimmune and miscellaneous conditions, represented 7.94% (25/315) of the cases, with a 16.67% adverse outcome rate (4/24). Significant variation existed within indirect factors for both distribution (χ² = 31.83, P < 0.001) and outcome rates (χ² = 9.26, P = 0.05). Overall, adverse outcome rates were statistically indistinguishable between direct (21.28%, 53/249) and indirect factors (21.21%, 14/66) (χ² = 0.36, P = 0.55) (Table 4).

**Table 4:** Distribution of Causes and Outcomes of Critical Maternal Cases in Wuxi City.

Cause Type	Specific Cause	Number of Cases (n)	Proportion of Concurrent Critical Cases (%)	Number of Adverse Outcomes (n)	Adverse Outcome Rate (%)	Intergroup Statistics (χ²/P-value)
Direct obstetric factors (total)	–	249	79.05	53	21.28	1. Differences in distribution compared to indirect obstetric factors: χ² = 89.62 / P < 0.001 2. Comparison with adverse outcome rates of indirect obstetric factors: χ² = 0.36 / P = 0.55
	Obstetric hemorrhage	170	53.97	36	21.17	1. Differences in the distribution of causes within the direct obstetric factors subgroup: χ² = 82.45 / P < 0.001 2. Comparison of adverse outcome rates within direct obstetric factors: χ² = 14.79 / P = 0.001
	Gestational Hypertensive Disorders	75	23.81	14	18.67	–
	Other (Amniotic Fluid Embolism)	4	1.27	3	75.00	–
Indirect Obstetric Factors (Total)	–	66	20.95	14	21.21	1. Differences in the distribution of underlying causes within indirect obstetric factors: χ² = 31.83 / P < 0.001 2. Comparison of adverse outcome rates within indirect obstetric factors: χ² = 9.26 / P = 0.05
	Cardiovascular Disease	19	6.03	5	26.32	
	Infectious Diseases	13	4.13	3	23.07	
	Acute Pancreatitis	6	1.90	1	16.67	
	Pneumonia	3	0.95	1	33.33	1. Differences in risk level distribution within the cycle: χ² = 28.95 / P < 0.001 2. Association between risk level and adverse outcomes within the cycle: χ² = 9.03 / P = 0.03
	Other (autoimmune diseases, etc.)	25	7.94	4	16.00	
Total	–	315	100	67	21.27	1. Differences in the distribution of all-cause types: χ² = 118.76 / P < 0.001 2. Comparison of adverse outcome rates across all-cause types: χ² = 18.35 / P < 0.01

Fetal sex was not assessed as a potential factor in this study, as the surveillance database did not routinely collect this information. While some studies have suggested associations between fetal sex and certain pregnancy complications (e.g., male fetus linked to higher risk of gestational diabetes or preterm birth), the evidence remains inconclusive, and the biological mechanisms are not well established. Given the descriptive and surveillance-oriented nature of our study, we focused on maternal characteristics and clinical factors directly related to obstetric care quality. However, given the predominance of obstetric hemorrhage and cardiovascular disease in our cohort, future studies could explore whether fetal sex modifies the risk or clinical presentation of these specific conditions. Incorporating fetal sex as a potential covariate may provide additional insights into the pathophysiology of severe maternal morbidity.

### Distribution of clinical interventions

Respiratory support (≥ 60 min) was the most frequent intervention (62.86%, 198 cases), followed by blood transfusion therapy (45.08%, 142 cases). Hysterectomy (n = 15) occurred exclusively in the direct factors group (6.02%). Renal dialysis and anti-infective therapy were significantly more common in the direct factors group (12.12% vs. 4.02%, P < 0.001) and (48.48% vs. 16.47%, P < 0.001), respectively (Table 5).

**Table 5:** Distribution of Clinical Treatment Measures for Critically Ill Pregnant Women.

Treatment Measure	Number of Cases (n)	Proportion of Total Critical Cases (%)	Direct Obstetric Factors Group (Cases/Percentage)	Indirect Obstetric Factors Group (Number of Cases/Percentage)
Respiratory Support (≥60 min)	198	62.86	156/62.65%	42/63.64%
Transfusion Therapy	142	45.08	118/47.39%	24/36.36%
Cardiopulmonary resuscitation	48	15.24	39/15.66%	9/13.64%
Kidney Dialysis	18	5.71	10/4.02%	8/12.12%
Hysterectomy	15	4.76	15/6.02%	0/0.00%
Anti-infective therapy	73	23.17	41/16.47%	32/48.48%

### Pregnancy outcomes by etiology

Pregnancy outcomes differed significantly between groups. The direct factor group demonstrated higher live birth rates (90.36% vs. 81.82%, χ² = 4.21, P = 0.04) and lower stillbirth rates (8.43% vs. 18.18%, χ² = 7.85, P = 0.005). The indirect factors group required earlier pregnancy termination (<28 weeks: 16.67% vs. 6.02%) and exhibited higher neonatal asphyxia rates (4.17% vs. 2.39%). Cesarean delivery was the predominant mode overall (71.11%), with no significant difference between groups (72.30% vs. 66.67%, χ² = 1.36, P = 0.24) (Table 6).

**Table 6:** Detailed Distribution of Pregnancy Outcomes Among Critically Ill Pregnant Women.

Outcome Indicator	Outcome Indicator	Number of Cases (n)	Proportion of Concurrent Critical Cases (%)	Direct Obstetric Factors Group (n/Percentage)	Indirect Obstetric Factors Group (n/Proportion)	Intergroup Comparison (χ² Value)	Intergroup Comparison (P-value)
Delivery Details	Live Birth	279	88.57	225/90.36%	54/81.82%	4.21	0.04
	Stillbirth	33	10.48	21/8.43%	12/18.18%		
	Deaths without termination of pregnancy	1	0.32	0/0.00%	1/1.52%	–	
Gestational age at termination	<28 weeks	26	8.25	15/6.02%	11/16.67%	7.85	0.0005
	28-37 weeks	203	64.44	14	168/67.47%		
	≥37 weeks	84	26.67	64/25.70%	20/30.30%		
Mode of Delivery	Vaginal delivery	89	28.25	67/26.91%	22/33.33%	1.36	0.24
	Cesarean section	224	22/33.33%	22/33.33%	44/66.67%		
	Induced Abortion/ Abortion	2	0.63	2/0.80%	0/0.00%		

## Discussion

This study identified a distinct dynamic pattern of critical maternal morbidity in Wuxi, characterized by three cycles of stability, followed by a significant surge in the fourth cycle. The incidence increased to 0.24% during the October 2023–September 2024 period (P = 0.02), which was positively associated with an increase in the proportion of pregnant women of advanced maternal age to 32.22% (χ² = 11.76, P = 0.001). Previous studies have established that advanced maternal age is independently associated with a higher risk of multiple pregnancy complications, with pre-existing comorbidities such as chronic hypertension and diabetes occurring 2–3 times more frequently than in pregnancies at the optimal reproductive age [24,25]. Physiological adaptations to pregnancy, including a 30% − 40% increase in blood volume and elevated cardiac output, further exacerbate organ system load, potentially leading to decompensation of underlying conditions and indirectly contributing to the observed rise in critical cases [26,27]. Notably, the increasing incidence may not solely reflect a true increase in the disease burden. As a major medical hub in the Yangtze River Delta region, Wuxi established a provincial-level critical maternal surveillance system and multidisciplinary case review mechanism in 2020, which significantly improved the sensitivity of case identification and the completeness of reporting [28]. Conditions such as early-stage heart failure and occult postpartum hemorrhage, which were previously underrecognized due to diagnostic delays or referral gaps, are now more reliably captured within the enhanced monitoring network. This suggests a positive trend in which the rising incidence coexists with an improved prognosis. Furthermore, compared with the reported incidence of 0.33% in central and western China [29], Wuxi’s rate of 0.233% remains relatively low, supporting the concept that optimized healthcare resources may reduce the severity threshold for the identification of critical maternal conditions. To assess whether the increasing trend in SMM incidence was driven by changes in transfusion practices, we performed a sensitivity analysis excluding cases where blood transfusion was the sole criterion for SMM. The incidence remained stable across the first three cycles (0.18%–0.19%) and increased to 0.23% in the final cycle (χ² = 4.89, P = 0.03), indicating that the observed trend is not solely attributable to transfusion practices.

A key finding of this study was the shift in risk-level distribution from a predominance of red (high-risk) cases to a rising prominence of moderate-risk cases. In earlier surveillance cycles, red-level cases accounted for 63.29% of critical maternal morbidity, whereas in recent cycles, moderate-risk cases (yellow and orange combined) increased to 64.44%, surpassing the proportion of high-risk cases for the first time. This trend differs from the patterns reported in resource-constrained settings, such as those described by Adeniran et al. in certain African regions, where limited healthcare access has historically led to a concentration of high-risk presentations [30]. The decline in the proportion of high-risk cases (from 63.29% to 32.22%) likely reflects the effectiveness of structured management protocols for conditions such as severe preeclampsia and placenta previa, which are managed through a coordinated pathway spanning pre-pregnancy assessment, enhanced antenatal monitoring, and planned early delivery [31,32]. Through multidisciplinary collaboration involving obstetrics, anesthesiology, and intensive care, and standardized interventions, adverse outcome rates in these high-risk cases have been maintained at a relatively low level (15.91%–20.69%). This supports the clinical principle that interventions in high-risk pregnancies can lead to controllable outcomes. In contrast, the moderate-risk group exhibited a persistently higher adverse outcome rate (25.00%–25.71%) than the high-risk group, highlighting a critical gap in the current maternal health care system. Indirect obstetric factors accounted for 43.15% (63/146) of moderate-risk cases, often involving conditions such as cardiac disease and infections, which may follow an insidious clinical course and exert systemic effects on the mother. Unlike high-risk pregnancies, which trigger intensive management, moderate-risk cases are often managed with routine prenatal care without mechanisms for dynamic risk escalation. For example, pregnant women with gestational diabetes who repeatedly fail to meet glycemic targets may not be promptly reclassified from yellow to orange risk, increasing their risk of progression to ketoacidosis [22,33]. This mismatch between intervention intensity and actual disease severity likely contributes to the higher rate of adverse outcomes in this group. Therefore, the increase in moderate-risk cases should not be interpreted as a failure of the risk assessment system but rather as an indicator of the need for more refined and responsive management strategies tailored to this population.

Direct obstetric factors remained the leading cause of critical maternal morbidity, accounting for 79.05% of the cases. However, indirect obstetric factors increased to 20.71%, with an increasing annual trend (from 17.81% to 26.71%), representing a growing challenge to regional obstetric safety. Among the direct causes, obstetric hemorrhage was the most prevalent (53.97%). Its multifactorial etiology, often involving placental disorders, uterine atony, or trauma to the birth canal, necessitates complex and tailored clinical management [34,35]. Consistent with this, the direct factor group in this study demonstrated significantly higher rates of blood transfusion (47.39%) and peripartum hysterectomy (6.02%) than the indirect factor group, underscoring the critical role of structured postpartum hemorrhage emergency protocols. Although amniotic fluid embolism was rare (1.27%), it was associated with a notably high adverse outcome rate (75.00%). Rapid clinical deterioration, often involving acute respiratory distress syndrome (ARDS) and disseminated intravascular coagulation (DIC), requires well-coordinated multidisciplinary management involving obstetrics, anesthesiology, and transfusion services [36]. Among indirect obstetric factors, cardiac disease was the most frequent (6.03%) and was associated with the highest adverse outcome rate (26.32%) across all etiologies. Physiological adaptations to pregnancy, including a 30–40% increase in blood volume and a 15–20% rise in heart rate, can increase the risk of cardiac dysfunction by 3- to 5-fold [37]. In line with these pathophysiological challenges, our analysis showed that the indirect factor group underwent earlier pregnancy termination (< 28 weeks: 16.67% vs. 6.02%, P = 0.005) and had a lower live birth rate (81.82% vs. 90.36%, P = 0.04). These findings are consistent with those reported by Ashrafi et al., indicating elevated maternal mortality in pregnancies complicated by cardiac disease [38].

This study delineates distinct etiology-driven patterns in the clinical management of critically ill obstetric patients. Respiratory support (62.86%) and transfusion therapy (45.08%) were the most common interventions, reflecting the high burden of respiratory failure and hemorrhagic shock in this critically ill obstetric population. The use of antimicrobial therapy was significantly more frequent in women with indirect obstetric factors (48.48% vs. 16.47%, P < 0.001), consistent with the higher prevalence of infectious etiologies and their associated risk of sepsis in this group [39]. Furthermore, renal dialysis was required in 12.12% of indirect-factor cases, suggesting a greater propensity for multiple organ dysfunction syndrome (MODS). The overall cesarean section rate was 71.11%, which aligns with international data on the delivery mode in critically ill pregnant women [40]. Although the rate was numerically higher in the direct factors group (72.30% vs. 66.67%), this difference was not statistically significant, a finding that may be influenced by sample size limitations and diverse obstetric indications across etiologies. Notably, the adverse outcome rate was higher in the moderate-risk (yellow/orange) group than that in the high-risk (red) group. Several hypotheses may explain this paradoxical finding. First, a form of collider bias (or selection bias) could be at play: women without overt risk factors (i.e., moderate-risk) who develop severe complications may represent a subgroup with unmeasured vulnerabilities or atypical disease progression, leading to delayed recognition and worse outcomes. Second, the intensive surveillance and proactive interventions routinely applied to high-risk pregnancies (e.g., early referral, closer monitoring) may effectively mitigate adverse outcomes, whereas moderate-risk pregnancies receive less intensive management despite harboring potential for rapid deterioration. This interpretation aligns with the concept of ‘prevention paradox’ in obstetric care, where the majority of adverse events arise from the larger, lower-risk population rather than the smaller, high-risk group. Our findings underscore the need for dynamic risk assessment tools that can detect subtle signs of decompensation in moderate-risk women and trigger timely escalation of care.This finding suggests that current risk-level classifications may inadvertently create a false sense of security, leading to intervention strategies that are not commensurate with the actual clinical severity in moderate-risk patients. To address this gap, we propose that moderate-risk pregnancies be designated as a “priority monitoring cohort.” Within this framework, electronic medical record systems can be leveraged to automatically trigger risk escalation and intensify prenatal surveillance upon the detection of predefined warning signs, such as hypertension with proteinuria [41].

Several limitations should be acknowledged. First, the study period (2020–2024) overlapped with the COVID-19 pandemic; however, only a minority of infectious cases were attributed to SARS-CoV-2, and exclusion of these cases did not alter the main findings (data not shown).Second, due to the design of the surveillance system, we did not collect data on potential confounders such as fetal sex, maternal anemia (unless severe enough to require transfusion), or altitude of residence, which have been associated with adverse pregnancy outcomes in some studies. Regarding anemia, mild-to-moderate cases are common in pregnancy but do not meet the provincial criteria for “critical maternal case” reporting; therefore, their contribution to SMM could not be assessed. High-altitude pregnancy was not relevant to this cohort, as Wuxi is a low-altitude region (approximately 10 meters above sea level) and migration patterns during the study period did not include residents from high-altitude areas. Future research incorporating these variables could provide a more comprehensive understanding of SMM determinants.

## Conclusion

This study presents a systematic analysis of 315 cases of SMM identified from 156,435 deliveries in Wuxi (October 2020–September 2024). The overall SMM incidence was 0.20%, which remained stable before rising significantly to 0.24% in the final surveillance cycle. This increase was associated with a growing proportion of women of advanced maternal age and improved case identification following enhancements in emergency obstetric care. We observed a marked shift in risk distribution: moderate-risk cases became predominant (43.59%) and exhibited significantly higher adverse outcome rates (25.00–25.71%) than the high-risk group (17.72%), identifying them as a critical priority for intervention. Direct obstetric factors were the primary etiology (79.05%), predominated by obstetric hemorrhage (53.97%). Indirect factors (20.95%) showed an upward trend, primarily attributable to cardiovascular disease (6.03%) and infections (4.13%). Their adverse outcome rate (21.21%) approached that of direct causes (20.88%), establishing indirect factors as an emerging challenge in the field. The etiology dictated distinct clinical patterns: direct SMM commonly required blood transfusions and hysterectomies, whereas indirect SMM necessitated renal dialysis and anti-infective therapy. The latter was also associated with an earlier gestational age at delivery and lower live birth rates. The study limitations include the limited generalizability of single-center data, small sample sizes for specific etiologies, and incomplete adjustment for socioeconomic confounders. Future studies should utilize multicenter designs with larger sample sizes to enable robust identification of risk factors. Clinical and public health initiatives should prioritize refining dynamic risk assessment, strengthening collaboration between obstetrics and internal medicine, and advancing integrated care from preconception to postpartum. These measures are essential for reducing adverse maternal and neonatal outcomes and improving the regional quality of obstetric care.

## Supporting information

S1 Data Supplementary material associated with this article can be found in Supporting information S1_Data. (XLSX)
